# Developing global indicators for quality of maternal and newborn care: a feasibility assessment

**DOI:** 10.2471/BLT.16.179531

**Published:** 2017-03-13

**Authors:** Barbara Madaj, Helen Smith, Matthews Mathai, Nathalie Roos, Nynke van den Broek

**Affiliations:** a Centre for Maternal and Newborn Health, Liverpool School of Tropical Medicine, Pembroke Place, Liverpool, L3 5QA, England.; b Department of Maternal, Newborn, Child and Adolescent Health, World Health Organization, Geneva, Switzerland.

## Abstract

**Objective:**

To assess the feasibility of applying the World Health Organization’s proposed 15 indicators of quality of care for maternal and newborn health at health-facility level in low- and middle-income settings.

**Methods:**

Six of the indicators are about maternal health, five are for newborn health and four are general cross-cutting indicators. We used data collected routinely in facility registers and obtained as part of facility assessments from 963 health-care facilities specializing in maternity services in 10 countries in Africa and Asia. We made a feasibility assessment of the availability of data and the clarity of indicator definitions and identified additional information and data collection processes needed to apply the proposed indicators in real-life settings.

**Findings:**

Of the indicators evaluated, 10 were clearly defined, of which four could be applied directly in the field and six would require revisions to operationalize them. The other five indicators require further development, with one of them being ready for implementation by using information readily available in registers and four requiring further information before deployment. For indicators that measure coverage of care or availability of services or products, there is a need to further strengthen measurement. Information on emergency obstetric complications was not recorded in a standard manner, thus limiting the reliability of the information.

**Conclusion:**

While some of the proposed indicators can already be applied, other indicators need to be refined or will need additional sources and methods of data collection to be applied in real-world settings.

## Résumé

**Objectif:**

Évaluer s'il est faisable d'appliquer les 15 indicateurs proposés par l'Organisation mondiale de la Santé relatifs à la qualité des soins de santé maternelle et néonatale dans des centres de santé de régions à revenu faible et intermédiaire.

**Méthodes:**

Six indicateurs portent sur la santé maternelle, cinq sur la santé néonatale et quatre sur des facteurs transversaux d'ordre général. Nous avons utilisé les données consignées habituellement dans les registres des centres de santé et obtenues dans le cadre d'évaluations de ces centres; des données couvrant au total 963 centres de santé, spécialisés dans les services de maternité, dans 10 pays d'Afrique et d'Asie. Nous avons réalisé une étude de faisabilité portant sur la disponibilité des données et la clarté des définitions des indicateurs, et nous avons identifié les processus de collecte des données et informations complémentaires nécessaires pour pouvoir appliquer ces indicateurs en conditions réelles.

**Résultats:**

Parmi tous les indicateurs évalués, 10 sont clairement définis, parmi lesquels quatre pourraient être directement appliqués sur le terrain, tandis que les six autres nécessiteraient des ajustements avant de pouvoir être mis en œuvre. Les cinq indicateurs restants nécessitent d'autres développements; même si l'un d'eux pourrait être exploité en utilisant les informations consignées dans les registres déjà disponibles, pour les quatre autres indicateurs, des informations complémentaires sont nécessaires. Pour les indicateurs relatifs à la couverture des soins ou à la disponibilité des services ou des produits, des évaluations plus détaillées seraient nécessaires. Les informations sur les complications obstétricales d'urgence n'ont pas été enregistrées de façon standardisée, ce qui limite la fiabilité de ces informations.

**Conclusion:**

Alors que certains des indicateurs proposés sont d'ores et déjà applicables, d'autres indicateurs doivent être affinés ou nécessitent des sources d’information supplémentaires et des méthodes complémentaires de collecte de données avant de pouvoir être appliqués en conditions réelles.

## Resumen

**Objetivo:**

Evaluar la viabilidad de aplicar los 15 indicadores de calidad de atención sanitaria materna y obstétrica propuestos por la Organización Mundial de la Salud en centros sanitarios de entornos con ingresos bajos y medios.

**Métodos:**

Seis de los indicadores tratan sobre salud materna, cinco sobre salud obstétrica y cuatro son indicadores transversales generales. Se utilizaron datos recopilados de forma rutinaria en los registros de los centros y se obtuvieron como parte de las evaluaciones de las instalaciones de 963 centros sanitarios especializados en servicios de maternidad de 10 países de África y Asia. Se realizó una evaluación de viabilidad sobre la disponibilidad de datos y la claridad de las definiciones de los indicadores, y se identificó información adicional y los procesos de recopilación de datos necesarios para implementar los indicadores propuestos en centros reales.

**Resultados:**

De los indicadores evaluados, 10 se definieron con claridad, de los cuales cuatro podían aplicarse directamente en el campo y seis necesitarían revisiones para hacerlos operativos. Los otros cinco indicadores requieren un mayor desarrollo: uno de ellos está listo para ser implementado utilizando información inmediatamente disponible en los registros y cuatro necesitan más información antes de su puesta en práctica. Para los indicadores que miden la cobertura de la atención o la disponibilidad de productos o servicios, se necesita fortalecer más la medición. No se ha registrado información sobre complicaciones obstétricas de emergencia de forma estándar, por lo que la fiabilidad de la información es limitada.

**Conclusión:**

Mientras que algunos de los indicadores propuestos ya pueden aplicarse, otros necesitan perfeccionarse o requerirán fuentes y métodos de recopilación de datos adicionales para poderse aplicar en entornos reales.

## ملخص

**الغرض:**

تقييم جدوى تطبيق 15 مؤشرًا مقترحًا لمنظمة الصحة العالمية لقياس جودة الرعاية الصحية للأمهات وحديثي الولادة على مستوى المرافق الصحية والمواقع ذات الدخل المنخفض والمتوسط.

**الطريقة:**

تتعلق ستة مؤشرات بصحة الأمومة، فيما تتعلق خمسة مؤشرات بصحة الأطفال حديثي الولادة، فضلاً عن أربعة مؤشرات عامة وشاملة. وقد استخدمنا البيانات المجمعة بصورة روتينية في سجلات المرافق الصحية والتي تم الحصول عليها كجزء من تقييمات المرافق المأخوذة من 963 مرفقًا للرعاية الصحية متخصصة في خدمات الأمومة في 10 دول في أفريقيا وآسيا. وقمنا بإجراء تقييم جدوى لمدى إتاحة البيانات ووضوح تعريفات المؤشر، وقمنا بتحديد عمليات إضافية لجمع البيانات والمعلومات سيتكون لازمة لتطبيق المؤشرات المقترحة على أرض الواقع.

**النتائج:**

من بين المؤشرات التي خضعت للتقييم، كان منها 10 مؤشرات تحمل تعريفات دقيقة، وكان من الممكن تطبيق أربعة مؤشرات منها مباشرةً على أرض الواقع، فيما تحتاج ستة منها إلى المراجعة قبل نقلها إلى مرحلة التنفيذ العملي. وتتطلب المؤشرات الخمسة الأخرى المزيد من التطوير، مع وجود أحد المؤشرات في حالة جاهزة للتطبيق باستخدام المعلومات المتوفرة بسهولة في السجلات، وأربعة مؤشرات بحاجة إلى المزيد من المعلومات قبل التنفيذ. بالنسبة للمؤشرات التي تقيس تغطية الرعاية أو مدى توافر الخدمات أو المنتجات، هناك حاجة إلى تعزيز القياس بشكل أكبر. لم يتم تسجيل المعلومات المتعلقة بمضاعفات الولادة في الحالات الطارئة بشكلٍ قياسي، مما حد من مدى اعتمادية المعلومات.

**الاستنتاج:**

بالرغم من إمكانية تطبيق بعض المؤشرات المقترحة بالفعل، فإن المؤشرات الأخرى بحاجة إلى إدخال التحسينات عليها أو قد تحتاج إلى مصادر وطرق إضافية لجمع البيانات ليتسنى تطبيقها في الواقع.

## 摘要

**目的:**

旨在评估在中低收入国家卫生机构实施世界卫生组织提出的 15 个孕产妇和新生儿健康质量指标的可行性。

**方法:**

其中六个指标与孕产妇健康相关，五个与新生儿健康相关，四个是一般性综合指标。 我们使用定期从医疗机构登记处收集的数据，以及从 10 个亚非国家 963 个专门提供孕产服务的医疗机构中获取的数据作为机构评估的一部分。 我们对数据的可用性以及指标定义的清晰度进行了可行性评估，同时明确了将所提议指标应用于现实环境所需的其他信息和数据收集流程。

**结果:**

在评估的指标中，10 个定义明确，其中，4 个可直接应用到实际中，6 个需要修订以便于实施。 其他五个指标需要进一步制定，其中一个可通过使用登记处的现有信息投入实施，另外四个在部署之前，需要增添一些补充信息。 对于测量护理覆盖率或者服务或产品可用性的指标，有必要进一步加强测量。 有关紧急产科并发症的信息未采用标准形式记录，因此影响了信息的可靠性。

**结论:**

虽然提出的有些指标已能够实施，但是其他指标仍需要改进或者需要额外的数据收集资源和方法，以便应用于现实环境中。

## Резюме

**Цель:**

Дать оценку практической возможности применения 15 предложенных Всемирной организацией здравоохранения показателей качества ухода за матерью и новорожденным ребенком в медицинских учреждениях в условиях с низким и средним уровнем доходов.

**Методы:**

Шесть показателей из них относятся к охране здоровья матери, пять — к охране здоровья новорожденного ребенка, и четыре являются показателями общего характера. Авторы статьи использовали данные, собранные в рабочем порядке в реестрах медицинских учреждений и полученные в ходе оценок работы медицинских учреждений, из 963 медико-санитарных учреждений, специализирующихся на оказании услуг по уходу за матерями, в 10 странах Африки и Азии. Была дана оценка практической возможности получения данных и точности формулировок определений показателей. Были определены дополнительные процессы сбора информации и данных, необходимые для применения предложенных показателей на практике.

**Результаты:**

Из всех показателей, подвергнутых оценке, определения десяти были сформулированы точно, и четыре из них могли быть применены непосредственно на практике, а шесть показателей необходимо скорректировать, прежде чем их можно будет практически реализовать. Остальные пять показателей требуют доработки, причем один из них может быть применен с использованием имеющейся в реестрах информации, а для остальных четырех необходима дополнительная информация, прежде чем их можно будет реализовать. Для показателей, используемых для измерения охвата медицинской помощью или доступности услуг или продуктов, требуется дополнительно усовершенствовать процесс измерения. Информация о неотложной акушерской помощи при осложнениях не была зафиксирована в установленном порядке, и это сказалось на ее надежности.

**Вывод:**

Хотя некоторые из предложенных показателей уже могут быть применены, для других показателей требуется доработка или дополнительные источники данных и методы их сбора, чтобы показатели можно было применить на практике.

## Introduction

Improving the quality of care for maternal and newborn health is important if health outcomes for mothers and babies are to continue to improve. This will require a renewed global focus.^1^ According to 2015 estimates, annually 303 000 women die during pregnancy, childbirth or in the postnatal period, 2.6 million babies are stillborn and 2.7 million babies die within 1 month of birth.^2^^–^^4^ The majority of these deaths occur in low- and middle-income settings and are preventable. Ensuring quality care is provided to every mother and newborn during this period is critical for maternal and newborn survival.

Monitoring of progress towards the achievement of millennium development goals 4 and 5 – i.e. reduce child mortality and improve maternal health, respectively – focused initially on measurement of coverage of evidence-based cost-effective interventions such as antenatal care and skilled birth attendance rates.^5^ Although intervention coverage rates (i.e. the number of people receiving an intervention or service from among those who need it) have been increasing rapidly,^6^^,^^7^ it is widely acknowledged that the quality of care provided for mothers and babies is rarely evidence-based and women-centred. Uptake (and coverage) of care and quality of care are also linked; numerous examples exist in the literature describing where and how poor quality of care has deterred women from accessing services even where these were available, close by and affordable.^8^^–^^11^

The United Nations (UN) Commission on Information and Accountability for Women and Children’s Health was established in 2011 to enhance local, national and global accountability for women and children’s health. The commission identified 10 recommendations to be adopted by countries.^12^ The first set of recommendations focused on better information for results, calling for countries to strengthen vital registration and health information systems, to focus on a core set of harmonized maternal newborn and child health indicators, and to invest in information, communication and technology to strengthen their national health information systems at all levels. More recently, Countdown to 2015 reports acknowledged the need to monitor quality of care as well as coverage of interventions, and the corresponding need for better data to do this.^13^ These developments are also identified in the sustainable development goals, with recognition that reaching such targets as reducing the maternal mortality ratio to under 70 per 100 000 live births by 2030, will require continued efforts to improve quality of care, underpinned by the availability of robust evidence.^14^^–^^18^

In response to the need for more and better data, the World Health Organization (WHO) was asked to propose trace indicators for quality of maternal and newborn health care provided at health-care facility level that could be used for global comparisons. After consultation with a wide range of international stakeholders and experts in quality of care, a core set of 15 indicators was proposed (Box 1).^19^ The indicators were thought to be good markers of lifesaving interventions and were obtained via consensus, but have not been formally assessed to determine whether they complement or link to data already routinely collected for maternal and newborn health.

Box 1 **Indicators for assessing quality of maternal and newborn health services proposed by the World Health Organization and stakeholders^a,b^**

**Maternal health**

M1: Proportion of antenatal care visits at which blood pressure was measured

M2: Proportion of women with severe pre-eclampsia or eclampsia treated with magnesium sulfate injection

M3: Proportion of women receiving oxytocin within 1 min of birth of infant

M4: Proportion of women with prolonged labour

M5: Intrapartum stillbirth rate

M6: Proportion of women with severe systemic infection or sepsis in postnatal period, including readmissions

**Newborn health**

N1: Proportion of health facilities with functional bags and masks (two neonatal mask sizes) in the delivery areas of maternity services

N2: Proportion of newborns who received all four elements of essential care:

- immediate and thorough drying
- immediate skin-to-skin contact
- delayed cord clamping
- initiation of breastfeeding in the first hour

N3: Proportion of health facilities in which kangaroo mother care is operational, by level of facility

N4: Facility neonatal mortality rate disaggregated by birth weight: > 4000 g, 2500–3999 g, 2000–2499 g, 1500–1999 g, < 1500 g

N5: Proportion of health facilities offering maternity services certified by the Baby-friendly Hospital Initiative and recertification no later than 2 years afterwards

**General indicators**

G1: Proportion of health facilities that have stock-outs of essential lifesaving medicines for mothers and newborns in a specified period

G2: Proportion of maternal and perinatal and child^c^ deaths occurring in a facility that were reviewed

G3: Proportion of health facilities with soap and running water or alcohol-based rub available in labour, childbirth, neonatal and paediatric wards

G4: Proportion of health facilities with safe, uninterrupted oxygen supply in childbirth, neonatal and paediatric wards

^a^ The abbreviations (M1, M2, etc.) are used to facilitate referencing in this paper and were not in the original report.^b^ This table excludes four child indicators proposed in the consultation^19^ because these were not part of this assessment.^c^ This analysis excludes the child death reviews.Source: World Health Organization, 2014.^19^

This paper describes a study of the feasibility of applying the proposed indicators in low- and middle-income settings. We assessed the availability of data in existing facility records and the clarity of indicator definitions, and identified additional information and processes needed to collect the data in real-life settings.

## Methods

### Data sources

For all indicators except one (G3), the information used in this evaluation was based on the results of a baseline facility assessment conducted by the Centre for Maternal and Newborn Heath at the Liverpool School of Tropical Medicine. This was part of a capacity-building programme implemented between January 2012 and December 2015 aiming to improve the availability and quality of emergency obstetric and newborn care (Making it Happen programme).^20^^,^^21^ A total of 963 health-care facilities in Bangladesh, Ghana, Kenya, Malawi, Nigeria, Pakistan, Sierra Leone, South Africa, the United Republic of Tanzania and Zimbabwe were surveyed (Table 1). All data collected for each facility referred to the quarter (3 months) immediately preceding the assessment. For the indicator on the proportion of health facilities with soap and running water or alcohol-based rub (G3) we used data from a facility survey in Sierra Leone, conducted as part of a study assessing the effect of the Ebola virus disease outbreak on availability, uptake and demand for essential maternal and newborn health services. Conducted in the month of February 2015, the survey included 76 facilities countrywide.

**Table 1:** Characteristics of the facilities and countries used in the feasibility analysis of proposed indicators for quality of maternal and newborn health services

Indicators assessed,^a^ by region and country	Country income level^b^	No. of areas surveyed^c^	No. (%) of health facilities sampled		
			All	Basic emergency obstetric care^d^	Comprehensive emergency obstetric care^e^
**All indicators, except G3**					
Asia					
Bangladesh	Lower-middle	7	49	25 (51)	24 (49)
Pakistan	Lower-middle	6	83	59 (71)	24 (29)
Subtotal	N/A	13	132	84 (64)	48 (36)
Africa					
Ghana	Lower-middle	3	106	52 (49)	54 (51)
Kenya	Lower-middle	6	279	214 (77)	65 (23)
Malawi	Low	1	69	61 (88)	8 (12)
Nigeria	Lower-middle	2	83	63 (76)	20 (24)
Sierra Leone	Low	14	67	63 (94)	4 (6)
South Africa^f^	Upper-middle	9	133	53 (40)	80 (60)
United Republic of Tanzania	Low	2	89	65 (73)	24 (27)
Zimbabwe^f,g^	Low	3	5	0 (0)	5 (100)
Subtotal	N/A	40	831	571 (69)	260 (31)
Total	N/A	53	963	655 (68)	308 (32)
**Indicator G3**^h^					
Africa					
Sierra Leone	Low	13	76	63 (83)	13 (17)

N/A: not applicable.

^a^ The indicators and their definitions were developed by the World Health Organization, 2014 (Box 1).^19^

^b^ Based on the World Bank country classification for 2015.^22^

^c^ For all countries, the areas and facilities for the surveys were selected by the respective ministries and represented geographical and administrative areas which were identified as needing capacity-building around emergency obstetric care. For indicator G3, the data came from a facility survey in Sierra Leone, conducted as part of a study assessing the effect of the Ebola virus disease outbreak on availability, uptake and demand for essential maternal and newborn health services.

^d^ Basic emergency obstetric care facilities are required to offer the following services: administer parenteral antibiotics, administer uterotonic drugs (i.e. parenteral oxytocin), administer parenteral anticonvulsants for pre-eclampsia and eclampsia (i.e. magnesium sulfate), manually remove the placenta, remove retained products (e.g. manual vacuum extraction, dilation and curettage), perform assisted vaginal delivery (e.g. vacuum extraction, forceps delivery) and perform basic neonatal resuscitation (e.g. with bag and mask).^23^

^e^ In addition to the seven services at basic level, comprehensive emergency obstetric care facilities are expected to provide blood transfusion services and perform surgery (e.g. caesarean section).^23^

^f^ South Africa data were not available for M1, M4, M6, N4 and G2; Zimbabwe data were not available for N4.

^g^ G3 is the proportion of health facilities with soap and running water or alcohol-based rub available in childbirth, neonatal and paediatric wards.

^h ^Only central-level referral hospitals were surveyed.

The facilities surveyed were selected by the respective ministries of health and included public health facilities designated to provide maternity services in purposively selected geographical and administrative areas (*n* = 53). With the exception of Zimbabwe, where only central-level referral hospitals were surveyed (*n* = 5), the sample included facilities offering either basic or comprehensive emergency obstetric care. This classification indicates the complexity of care provided (with comprehensive emergency obstetric care facilities being required to offer caesarean section and blood transfusion services in addition to basic package of care) and broadly serves as an indication of the facility size.^23^

The primary data, which were subsequently used in the feasibility assessment, were collected prospectively according to the respective programme protocol using a standardized tool incorporating elements from the WHO and Averting Maternal Death and Disability health facility assessment tools.^24^ Information was collected during health-facility visits by trained data collectors who interviewed health-care providers. Data were verified using routine facility data sources, e.g. labour ward and operating theatre registers, newborn care unit registers and patient discharge registers.

Permission to conduct the facility assessments was granted by the respective ministries of health. Our audit covered existing data that were already available in standard, anonymized records. No information which would compromise the confidentiality or privacy of patients or staff was recorded or included in the analysis.

### Data extraction and analysis

For this assessment we first identified and extracted the data required to measure each indicator. This enabled us to assess the availability of the information in routinely collected facility records and, where possible, the completeness of the records across countries. From discussions among the research team, who were experienced in conducting facility assessments, we examined the clarity of the indicator definitions against the information currently available in facility records. For indicators where no data were readily available, and based on the team’s clinical and research expertise, we assessed the feasibility of obtaining the necessary information. We also assessed the approach and methods needed to measure the indicator at the health-facility level, using alternative measures at facility level. Finally, in cases where the proposed indicator could not be assessed in full, we developed proxy measures for which routine data and data collection systems are readily available.

We present the assessment findings for each indicator by summarizing the descriptive information and by analysing the availability of the required data using descriptive statistics.

## Results

Table 2 provides a summary of all proposed indicators based on the clarity of definitions and the availability of routine information to assess each indicator. Overall, 10 of the 15 indicators were considered to be clearly defined in their current format. However, using available facility registers, data would in principle be immediately accessible only for four indicators (M5, N1, N5 and G3), while the other six (M1, M2, M3, N2, N3 and G4) would require additional sources of information to operationalize them. Among the five indicators which require some further development, one (G1) could be implemented with currently available information, while the remaining four (M4, M6, N4 and G2) would need supplementary information.

**Table 2:** Classification of proposed indicators for quality of maternal and newborn health services according to clarity of definitions and availability of information at health-care facility level

Clarity of indicator	Information readily available	Additional information required
Clearly defined	M5: Intrapartum stillbirth rate N1: Health facilities with functional bag and mask N5: Health facilities with Baby-friendly Hospital Initiative G3: Health facilities with soap and running water or alcohol-based rub	M1: Antenatal care visits with blood pressure measured M2: Women with severe (pre)eclampsia treated with magnesium sulfate M3: Women receiving oxytocin N2: Newborns receiving all elements of essential care N3: Health facilities with operational kangaroo mother care G4: Health facilities with uninterrupted oxygen supply
Requires specification or adapting	G1: Health facilities with stock-outs of essential drugs	M4: Women with prolonged labour M6: Women with severe systemic infection or sepsis N4: Newborn deaths disaggregated by weight G2: Maternal, perinatal and child facility deaths reviewed

^a^ Indicators were developed by the World Health Organization, 2014.^19^

We further analysed each proposed indicator with regard to its potential for application in real-world settings. The key findings regarding the availability of data for each indicator and discussion of alternative indicators or additional methods of assessing the indicator are summarized in Table 3 (available at: http://www.who.int/bulletin/volumes/95/6/16-179531). Table 4 (available at: http://www.who.int/bulletin/volumes/95/6/16-179531) shows the availability of data across countries. Country-specific differences were noted with regard to individual indicators. However, across all countries, emergency obstetric complications posed a challenge because existing registers lack dedicated space for recording cases and consequently information on complications is not recorded in a standard manner, thus limiting the reliability of the information. Additionally, on a more practical level, anecdotal evidence from fieldworker notes suggests assessments in larger facilities required consulting records and registers from various wards and sources and therefore took more time and effort to consolidate the findings. Data on the number of women giving birth and the number of babies born were available at all facilities.

**Table 3:** Assessment of the feasibility of applying the proposed World Health Organization indicators for quality of maternal and newborn health services

Indicator^a^	Required information^a^	Feasibility assessment				
		Indicator clearly defined	Information readily available	Information available in facilities	Feasibility summary^b^	Suggestions for testing, applying and refining indicator
M1: Proportion of antenatal care visits at which blood pressure was measured	Numerator: number of women with blood pressure measured at antenatal care visit Denominator: number of women attending antenatal care clinics	Yes	No	Proxy measure used: Availability of blood pressure monitors in maternity services Number of women attending antenatal clinics	Availability of blood pressure monitors was widely reported; data missing in 0–10% of facilities across countries Information from antenatal clinics was not assessed in the surveys but data on number of women having their blood tested at a visit is not likely to be captured in facility-based records, but will be recorded in patient notes	Pilot-testing of indicator should include: observation of practice in health facilities (recording number of patients with blood pressure measured at antenatal care visit); and analysis of a sample of patient notes (to assess the percentage of previous visits with blood pressure measured) The pilot test would be useful to calculate the sample size required for full testing of the indicator Comparison of services offered at booking (first) visit versus follow-up visits could be done to verify the standards over time Information on urine tests (protein levels) could be added to strengthen the indicator
M2: Proportion of women with severe pre-eclampsia or eclampsia treated with magnesium sulfate injection	Numerator: number of women with (pre)eclampsia treated with magnesium sulfate injection Denominator: number of women with (pre)eclampsia	Yes	No	Number of women giving birth Availability of magnesium sulfate Number of (pre)eclampsia cases	Data on number of women giving birth were recorded in 100% of facilities Number of (pre)eclampsia cases was generally reported; data missing in 0–31% facilities across countries Bangladesh, Nigeria and Ghana most affected by missing information Availability of magnesium sulfate was generally reported; data missing for 0–8% facilities across countries Whether patients are treated with magnesium sulfate was not recorded in registers	Assessing adherence to the standard would require: collecting additional information at facility level from patient notes; and observation of practice (difficult due to infrequency of cases) A pilot test would be useful to calculate the sample size required for full testing of the indicator A review of facility policy and guidelines as an additional proxy indicator would help evaluate the indicator
M3: Proportion of women receiving oxytocin within 1 minute of birth of infant	Numerator: number of women receiving oxytocin within 1 minute of birth of infant Denominator: number of women giving birth	Yes	No	Number of women giving birth Availability of oxytocin	Data on number of women giving birth were recorded in 100% of facilities Availability of oxytocin was generally reported; data missing in 0–9% of facilities across countries Data on women receiving oxytocin and time of administration were not available	Although oxytocin availability is reported in facility surveys, the indicator in its current format would not be obtainable without additional recording systems to allow for capturing the time aspect Feasibility of the indicator could be better assessed using observation of practice on labour ward and review of national or local guidelines for post-delivery oxytocin administration, especially with relation to timeframe Timeframe for administration of oxytocin should follow evidence-based recommendations; the existing protocols for active management of the third stage of labour are not prescriptive on the time, though Administration of oxytocin may not be feasible within 1 minute of birth; the time-limit specified in the indicator may need to be reviewed
M4: Proportion of women with prolonged labour	Numerator: number of women with prolonged labour Denominator: number of women giving birth	No	No	Number of women giving birth Proxy: number of ruptured uterus cases Use of partograph	Use of partographs was widely reported; data missing in 0–4% of facilities across countries Number of cases of ruptured uterus was generally reported; data missing in 0–27% of facilities across countries. Nigeria, Bangladesh and Ghana were most affected by missing data Data on obstructed labour cases in facilities were available but were deemed unreliable due to poor data recording and lack of an agreed standard for classifying the complication; therefore, availability of data was not included in this assessment	Clearer definition of prolonged labour is needed for the assessment of proportion of cases to be calculated Classification of prolonged labour may very between countries; a standardized definition is needed Assessing the quality of partograph completion (via retrospective review of partographs), not only frequency of use, would provide additional information on correct use In facilities without partographs, retrospective review of patient notes to assess the diagnosis could be applied Alternative indicator could be the proportion of deliveries monitored with partograph, among women delivering at the facility (the data need to take account of women being referred to a facility having started labour elsewhere; for referral cases complications may not necessarily reflect the standard of care in the referral facility) Consideration should be given to measuring the number of cases of ruptured uterus among women delivering at (but not referred to) the facility as a measure of quality of care offered at the facility
M5: Intrapartum stillbirth rate	Numerator: number of fresh stillbirths Denominator: number of births	Yes	Yes	Number of births Number of stillbirths including classification into fresh and macerated births	Data on number of births were available in 100% of facilities Information on the number of stillbirths was widely reported; data missing in 0–5% of facilities across countries Standard of reporting fresh and macerated stillbirths across facilities at country level (except in South Africa, where the disaggregation was not recorded); data were missing in 1–36% of cases. Bangladesh, Nigeria and Pakistan were most affected by missing information	Due to the potential limitations of recording systems and the risk of classifying stillbirths incorrectly it may be advisable to report total stillbirth rate instead of intrapartum stillbirth rate Data on fetal heart rate monitoring, as well as information on weight for babies who were stillborn, would be challenging to capture via routine care records and would require partograph review, as per indicator M4
M6: Proportion of women with severe systemic infection or sepsis in postnatal period, including readmissions	Numerator: number of women with severe systemic infection in postnatal period; number of women with sepsis in postnatal period; number of women with severe systemic infection readmitted; number of women with sepsis readmitted Denominator: number of women giving birth	No	No	Number of women giving birth Number of postnatal sepsis cases (per quarter) Availability of antibiotics: penicillin, metronidazole, gentamicin and cephalosporin	Data on number of women giving birth were recorded in 100% facilities Number of postnatal sepsis cases was recorded in registers for current patients only; data missing in 1–27% of facilities across countries. Nigeria and Ghana were most affected by the lack of information Proxy: Data on availability of antibiotics were reported (see indicator G1) Data on readmissions were not easily available and no system for linking cases from delivery to readmission was identified in facilities (other than through individual case notes)	Definition needs to specify the population of women considered (only those women delivering in a facility or also those admitted after home birth or birth in a different facility) and to include guidelines for diagnosing severe systemic infection (e.g. standards and/or protocols for monitoring temperature as a symptom of infection, identifying sepsis) Specific guidelines for diagnosing severe systemic infection (e.g. fever as an infection, sepsis) are needed to operationalize the indicator Linking patients in records from original record to readmission may be challenging; use of patient notes instead may allow for linking of patient-specific information Calculating the number of women with sepsis post-delivery as a proportion of all deliveries at facility level would be a useful measure of quality of care
N1: Proportion of health facilities with functional bags and masks (two neonatal sizes) in the delivery areas of maternity services	Numerator: number of facilities with functional bag and mask (two neonatal mask sizes) available Denominator: number of facilities	Yes	Yes	Availability of bag and mask for neonatal resuscitation	Availability of bag and mask was widely reported; data missing in 0–4% of facilities across countries Data on mask sizes were not available	An additional indicator to assess the process for resuscitation of newborns would improve evaluation of quality of care
N2: Proportion of newborns who received all four elements of essential care	Numerator: number of newborns who received all four elements of essential care Denominator: number of live births	Yes	No	No proxy measures available	No standardized data were available at facilities (e.g. from case notes)	Observation of practice in labour ward is required to assess the feasibility of the indicator A review of regional or local policy guidelines would provide additional information on the standard applied at present Linking information on oxytocin use (indicator M3) could strengthen the evidence on quality of care
N3: Proportion of health facilities in which kangaroo mother care is operational, by level of facility	Numerator: number of facilities with operational kangaroo care Denominator: number of facilities	Yes	No	No proxy measures available	No data available	Although the standard for kangaroo mother care is clearly defined, the indicator would require a clearer definition of what constitutes operational kangaroo care An indicator based on whether a facility is able to offer kangaroo care may be more suitable Indicator could be measured by survey (including phone assessment) and as part of a standard facility assessment tool Information on use of kangaroo care is not collected in registers, therefore it would be necessary to verify how it is (or could be) recorded at facility level Staff may not know the criteria for kangaroo care or confuse it with skin-to-skin care. Examining available policy does not assess what is happening and how many babies who need kangaroo care actually receive it
N4: Facility neonatal mortality rate disaggregated by birth weight: > 4 000 g, 2 500–3999 g, 2000–2499 g, 1 500–1999 g, < 1 500 g	Numerator: number of neonatal deaths per weight category (> 4 000 g, 2500–3999 g, 2000–2499 g, 1 500–1999 g, < 1 500 g) Denominator: number of live births	No	No	Proxy: number of live births and number of babies discharged alive	Neonatal death rates could be calculated from difference between number of babies discharged alive and number of live births in the facility; data missing in 2–93% of facilities across countries. Only Sierra Leone and Kenya had < 10% facilities missing data Deaths were not reported by currently specified weight categories at facility level	Clearer definition of neonate is needed; if it is defined as up to 28 days the indicator will only capture information on babies still at the facility, and exclude those who die post-discharge, outside the facility. The indicator could specify that post-initial discharge or admissions to newborn care unit after home birth or birth in a different facility are to be included in the calculations An updated register with data on baby’s weight at time of discharge and on neonatal deaths (both regarding recording of neonatal deaths and the disaggregation by weight) is necessary Simpler weight categories might enable easier classification to distinguish between normal and low-birth-weight babies Standards for record-keeping need to include systematic data collection on neonatal deaths to allow for the indicator to be available in principle. When these data are available, weight categories may be included Indicator could be linked with indicator N3 on kangaroo care to provide a comprehensive assessment of quality of care
N5: Proportion of health facilities offering maternity services that are certified as Baby-Friendly	N/A	Yes	Yes	N/A	In many countries this information was only available at health facilities which had been part of a specific programme to introduce the Baby-Friendly accreditation,^25^ and the information was not collected for this assessment	N/A
G1: Proportion of facilities that had stock-outs of essential lifesaving medicines for mothers and newborns in a specified period	Numerator: number of facilities with essential lifesaving medicines for mothers and newborns available Denominator: number of facilities	No	Yes	Availability of antibiotics^c^ Availability of oxytocics^c^ Availability of anticonvulsants and antihypertensives^c^	Availability of medicines was generally well reported, although completeness of reporting varied across drug types; data missing in 0–19%, 0–9% and 0–8% for antibiotics, oxytocics and anticonvulsants, respectively, across countries	Clearer definition of stock-out is needed (drug not available at all or temporarily unavailable). Temporary unavailability needs to specify the number of days acceptable before being classified as stock-out List of essential drugs needs to be specified, taking into account regional or local guidelines and practices. Include a tracer drug or drugs (at least one of which needs to be available) to allow for standard monitoring Time period should be specified in the definition (e.g. per quarter), in line with UN facility survey standards
G2: Proportion of maternal and perinatal deaths occurring in a facility that were reviewed	Numerator: number of maternal and perinatal deaths reviewed Denominator: number of maternal, perinatal and child deaths	No	No	Number of deaths occurring in facility (maternal and perinatal) Number of death reviews in facility (maternal and perinatal) Proxy: Availability of quality improvement committee Proxy: Action taken after quality improvement committee meeting Proxy: Availability of maternal death audit Proxy: Action taken after maternal death audit Proxy: Availability of perinatal or stillbirth review Proxy: Action taken after perinatal and stillbirth review	Information on deaths occurring in facilities and corresponding reviews is collected in facilities, especially those with quality improvement activities. However, the information was not collected in facility surveys, and therefore a proxy was used in this assessment Availability of committees was widely reported; missing data in 0–15% of facilities across countries (missing data calculations were based on maximum number of facilities with missing data within each of three categories: quality improvement committee; maternal death reviews; and perinatal death reviews) Information on action taken following the reviews was less reliable and not captured in a standardized way in facilities; therefore, it was not presented in this assessment This assessment did not include child death reviews	Definition of indicator requires clarification, as child (i.e. under 5 years old) death review is not a standard facility-based audit (perinatal and maternal death audits are more common) Collecting facility-level data on deaths reviewed can only be done in facilities with relevant committees established Information on individual deaths and corresponding reviews requires a clear time reference (i.e. deaths which occurred in the current review period that were reviewed in that period, or deaths from the previous review period that were not reviewed at the time, or deaths that occurred in the current review period but not reviewed) Frequency of reviews is important to assess, but such information is not available in most countries. Until a higher level system is available, individual facility data could serve as a useful proxy, but requires a reporting tool for committee activities linked to registry data on recoded deaths
G3: Proportion of facilities with soap and running water or alcohol based rub available in childbirth, neonatal and paediatric wards	Numerator: availability of running water, availability of alcohol-based rub Denominator: number of facilities with labour, neonatal and paediatric wards	Yes	Yes	Availability of water Source of water (for facilities with water available) Availability of handwashing facilities Types of hand-cleaning agents available	Water availability was widely reported; data missing in 3% of facilities (2/76, all offering only basic emergency obstetric care services) Availability of handwashing facilities was widely reported; data missing in only 1 facility (1%) Data on alcohol-based rubs were not collected in the survey	Definition of running water is required Indicator is measurable through WASH questions (some of which are already included in facility assessment survey tools)^26^ Alternative indicator could be used to assess availability of steady supply of clean water and soap or alcohol-based rub Observation and review of policies or guidelines at facility level are additional ways to measure hand-washing standards
G4: Proportion of health facilities with safe, uninterrupted oxygen supply in childbirth, neonatal and paediatric wards	Numerator: number of facilities with safe and uninterrupted supply of oxygen in designated wards Denominator: number of facilities with labour, neonatal and paediatric wards	Yes	No	No proxy measures available	N/A	Data are not currently available in standard facility records, so it would be necessary to verify how they are (or could be) recorded at facility level Definition of standards covering safe and uninterrupted oxygen supply is required to standardize measurements Assessment could be included as part of standard facility assessment of drugs, equipment and supplies availability

N/A: data not available; UN: United Nations; WASH: water, sanitation and hygiene.

^a^ Indicators and their definitions were developed by the World Health Organization, 2014.^19^

^b^ Missing data are presented in full in Table 4. Countries and number of facilities included in the feasibility assessment were: Bangladesh, 49; Ghana, 106; Kenya, 279; Malawi, 69; Nigeria, 83; Pakistan, 83; Sierra Leone, 67; South Africa, 133; United Republic of Tanzania, 89; Zimbabwe, 5. For indicator G3, data were obtained from a separate survey of 76 facilities in Sierra Leone.

^c^ Based on data on the availability of selected essential medicines: antibiotics (penicillin, metronidazole, gentamicin, and cephalosporin), oxytocics (oxytocin, misoprostol), anticonvulsant (magnesium sulfate) and antihypertensive (nifedipine) over the period of the evaluation (3 months), with options to select always available, available with stock-outs and not available.

**Table 4:** Information available to assess proposed World Health Organization indicators for quality of maternal and newborn health services: missing data, by country

Indicator	Information assessed	No. (%) of facilities with missing data, by country									
		Bangladesh (*n* = 49)	Ghana (*n* = 106)	Kenya (*n* = 279)	Malawi (*n* = 69)	Nigeria (*n* = 83)	Pakistan (*n* = 83)	Sierra Leone (*n* = 67)	South Africa (*n* = 133)	United Republic of Tanzania (*n* = 89)	Zimbabwe (*n* = 5)
M1: Proportion of antenatal care visits at which blood pressure was measured	Availability of blood pressure monitors in maternity services	3 (6)	6 (6)	2 (1)	4 (6)	5 (6)	0 (0)	0 (0)	N/A	9 (10)	0 (0)
M2: Proportion of women with severe pre-eclampsia or eclampsia treated with magnesium sulfate injection	Availability of magnesium sulfate	1 (2)	5 (5)	8 (3)	1 (1)	4 (5)	0 (0)	5 (8)	1 (1)	2 (2)	0 (0)
	Number of (pre)eclampsia cases (per quarter)	15 (31)	18 (17)	12 (4)	0 (0)	20 (24)	2 (2)	1 (2)	14 (11)	4 (5)	0 (0)
M3: Proportion of women receiving oxytocin within 1 minute of birth of infant	Availability of oxytocin	2 (4)	6 (6)	4 (1)	1 (1)	4 (5)	1 (1)	1 (2)	1 (1)	8 (9)	0 (0)
M4: Proportion of women with prolonged labour	Use of partographs	0 (0)	3 (3)	3 (1)	0 (0)	3 (4)	0 (0)	1 (2)	N/A	0 (0)	0 (0)
	Number of cases of ruptured uterus	12 (25)	23 (22)	14 (5)	0 (0)	22 (27)	1 (1)	1 (2)	N/A	5 (6)	0 (0)
M5: Intrapartum stillbirth rate	Number of stillbirths	1 (2)	2 (2)	3 (1)	0 (0)	3 (4)	1 (1)	0 (0)	6 (5)	0 (0)	0 (0)
	Number of stillbirth cases disaggregated into fresh and macerated (per quarter)^a^	158/434 (36)	59/772 (8)	12/1339 (1)	3/406 (1)	158/487 (32)	66/339 (20)	3/70 (4)	1118/1118 (100)	3/420 (1)	0/545 (0)
M6: Proportion of women with severe systemic infection or sepsis in postnatal period, including readmissions	Number of postnatal sepsis cases (per quarter)	3 (6)	16 (15)	20 (7)	1 (1)	22 (27)	3 (4)	3 (5)	N/A	4 (5)	1 (20)
N1: Proportion of health facilities with functional bags and masks (two neonatal sizes) in the delivery areas of maternity services	Availability of bag and mask for neonatal resuscitation^b^	0 (0)	0 (0)	0 (0)	0 (0)	3 (4)	0 (0)	0 (0)	1 (1)	0 (0)	0 (0)
N2: Proportion of newborns who received all four elements of essential care	N/A	N/A	N/A	N/A	N/A	N/A	N/A	N/A	N/A	N/A	N/A
N3: Proportion of health facilities in which kangaroo mother care is operational, by level of facility	N/A	N/A	N/A	N/A	N/A	N/A	N/A	N/A	N/A	N/A	N/A
N4: Facility neonatal mortality rate disaggregated by birth weight	Number of live births and number of babies discharged alive^c^	5 (10)	63 (59)	26 (9)	64 (93)	50 (60)	33 (40)	1 (2)	N/A	43 (48)	N/A
G1: Proportion of facilities that had stock-outs of essential lifesaving medicines for mothers and newborns in a specified period	Availability of antibiotics^d^ (per quarter)	5 (10)	5 (5)	4 (1)	3 (4)	4 (5)	16 (19)	5 (8)	1 (1)	7 (8)	0 (0)
	Availability of oxytocics^d^ (per quarter)	2 (4)	6 (6)	6 (2)	1 (1)	4 (5)	1 (1)	2 (3)	1 (1)	8 (9)	0 (0)
	Availability of anticonvulsants and antihypertensives^d^ (per quarter)	2 (4)	8 (8)	8 (3)	1 (3)	5 (6)	1 (1)	5 (8)	1 (1)	6 (7)	0 (0)
G2: Proportion of maternal and perinatal deaths occurring in a facility that were reviewed	Availability of quality improvement committee^e^	0 (0)	1 (1)	3 (1)	0 (0)	3 (4)	0 (0)	2 (3)	N/A	13 (15)	0 (0)
	Availability of maternal death reviews^e^	0 (0)	0 (0)	3 (1)	1 (1)	3 (4)	0 (0)	3 (5)	N/A	6 (7)	0 (0)
	Availability of perinatal and stillbirth reviews^e^	1 (2)	1 (1)	3 (1)	0 (0)	3 (4)	0 (0)	0 (0)	N/A	4 (5)	0 (0)
G3: Proportion of facilities with soap and running water or alcohol based rub available in childbirth, neonatal and paediatric wards^f^	Availability of water	N/A	N/A	N/A	N/A	N/A	N/A	2/76 (3)	N/A	N/A	N/A
	Availability of handwashing facilities	N/A	N/A	N/A	N/A	N/A	N/A	1/76 (1)	N/A	N/A	N/A
G4: Proportion of health facilities with safe, uninterrupted oxygen supply in childbirth, neonatal and paediatric wards	N/A	N/A	N/A	N/A	N/A	N/A	N/A	N/A	N/A	N/A	N/A

N/A: not available.

^a^ Calculation based on proportion of all stillbirths recorded across all health facilities for which disaggregation into fresh and macerated was not available.

^b^ Data on mask sizes were not available.

^c^ Data disaggregated by weight categories were not available.

^d^ Missing data calculations were based on availability of selected essential medicines: antibiotics (penicillin, metronidazole, gentamicin, cephalosporin), oxytocics (oxytocin, misoprostol), anticonvulsants (magnesium sulfate) and antihypertensives (nifedipine) over the period of the evaluation (3 months), with options to select always available, available with stock-outs and not available. The table shows the maximum number of facilities with missing data on any of the medicines within the group.

^e^ Missing data calculations were based on maximum number of facilities with missing data within each category: quality improvement committee, maternal death reviews, and perinatal and stillbirth reviews.

^f^ Only assessed in Sierra Leone.

Note: Indicators were developed by the World Health Organization, 2014.^19^

Our surveys did not collect information for indicators M1, M4, M6, N4 and G2 in South Africa and indicator N4 in Zimbabwe, thus affecting the denominators used in calculations in the assessment.

### Maternal health indicators

M1: Proportion of antenatal visits at which blood pressure was measured. Data available for the assessment of the indicator did not include information from antenatal clinics. Instead, a proxy measure was derived using the availability of blood pressure monitors of any type in maternity services. Generally, data on the availability of these monitors were accessible at facilities and only 3% of facilities overall (29/830) could not provide the information (Table 3).

M2: Proportion of women with severe pre-eclampsia or eclampsia treated with magnesium sulfate injection. Data on women treated with magnesium sulfate were not routinely available. Instead, data on number of (pre)eclampsia cases and availability of magnesium sulfate were used as proxies. Overall, 9% of facilities (86/963) did not hold records on numbers of patients with (pre)eclampsia, with missing data most pronounced at country level in Bangladesh, Ghana and Nigeria (Table 4). Data on magnesium sulfate availability show that 3% of facilities (27/963) were not able to provide the information.

M3: Proportion of women receiving oxytocin within 1 min of birth of infant. Data on availability of oxytocin were widely available and missing in only 3% of facilities (28/963). However, the use of oxytocin as part of Active Management of the Third Stage of Labour (AMTSL) and/or whether AMTSL was practised was not routinely recorded in birth registers.

M4: Proportion of women with prolonged labour. Routine use of the partograph and number of cases of ruptured uterus were used as proxy measures for this indicator. All except 1% of facilities (10/830) were unable to provide data on partograph use. Recorded cases of ruptured uterus were missing in 9% of facilities (78/830), although there was variability between countries, with facilities in Bangladesh, Ghana and Nigeria facing challenges in reporting data (Table 4).

M5: Intrapartum stillbirth rate. Fresh stillbirth may be used as a surrogate measure for intrapartum stillbirths, although information on fetus weight at admission or whether fetal heart rate was heard was not generally available in facility registers. Data on stillbirths were widely available (2%, 16/963 facilities overall had missing data), but data with stillbirths disaggregated into fresh and macerated were missing for over a quarter of reported stillbirths (27%, 1580/5930). Except in South Africa, which does not report these data, disaggregation of stillbirths forms part of routine record-keeping; however, data were more commonly missing in Bangladesh, Nigeria and Pakistan (Table 4).

M6: Proportion of women with severe systemic infection or sepsis in postnatal period, including readmissions. Data on the number of postnatal sepsis cases were missing in 9% of facilities (73/830), but data on readmissions were not available in any health-facility registers.

### Newborn health indicators

N1: Proportion of health facilities with functional bags and masks (two neonatal mask sizes) in the delivery areas of maternity services. Data on bag and mask availability were largely accessible, with missing data at very few facilities (< 1%, 4/963) (Table 3). However, data on specific sizes of bag and masks were not available.

N2: Proportion of newborns receiving all four elements of essential care. This was not documented as part of any routine register in the surveyed health facilities.

N3: Proportion of health facilities in which kangaroo mother care is operational. Although, in principle, information on whether kangaroo mother care was provided was anecdotally available in facilities, this indicator was not assessed routinely or recorded in any existing register at facility level.

N4: Facility neonatal death rate disaggregated by birthweight. Based on the assessment, health facilities mostly lacked neonatal discharge and death registers. Moreover, no data on deaths by birth weight categories were available. Neonatal death rates could be estimated in 540/825 (66%) of all facilities surveyed, based on the difference between the numbers of babies discharged alive and number of live births in the facility. However, babies discharged alive comprised both babies born in the facility and those referred from outside, thus potentially limiting the usability of the data. It was, nevertheless, the only proxy measure available.

N5: Proportion of health facilities offering maternity services that are certified as baby-friendly under the Baby-Friendly Hospital Initiative.^25^ In many countries this information was only available at health facilities which had been part of a programme to specifically introduce this accreditation, and the information was not available from facility records.

### General indicators

G1: Proportion of facilities which had stock-outs of essential lifesaving medicines for mothers, newborns and children in a specified period. Medicines assessed in the health facility surveys covered only part of the WHO essential drugs list. Nevertheless, information was readily accessible on the availability (i.e. whether a particular drug was available at all times, with stock-outs or not at all in the 3 months covered by the survey) of selected antibiotics (penicillin, metronidazole, gentamicin, cephalosporin), oxytocics (oxytocin, misoprostol) and an anticonvulsant and antihypertensive drug (magnesium sulfate, nifedipine). Among the groups of drugs, the highest percentage of missing data for availability of individual medicines among antibiotics was at 4% (37/963), 3% (28/963) for oxytocics and 4% (31/830) for anticonvulsants (Table 3).

G2: Proportion of maternal, perinatal and child deaths occurring in a facility that were reviewed. Data on the proportion of deaths reviewed were not collected as part of the health-facility assessments. However, for maternal and perinatal deaths, availability of review committees and whether or not action was taken could be used as proxy indicators. Data on these quality improvement activities were largely available. The existence of a quality improvement committee was reported by all except 3% of facilities (22/830), while information on the existence of maternal death reviews and perinatal/stillbirth review was missing in 2% (16/830) and 1% (12/830) of facilities respectively. However, data on actions taken were not necessarily informative and lacked detail of what the action entailed, and no standardized system for reporting the information was identified.

G3: Proportion of health facilities with soap and running water or alcohol-based rub. The feasibility of this indicator was assessed only in Sierra Leone. Data on water availability were generally accessible, with data missing for 3% of facilities (2/76), both offering basic emergency obstetric care services (Table 4). Information on availability of hand-washing facilities was missing at just 1% of facilities (1/76), with details of products for hand-hygiene widely available.

G4: Proportion of health facilities with safe, uninterrupted oxygen supply in childbirth, neonatal and paediatric wards. Data necessary to inform the indicator were not routinely collected at facility level.

## Discussion

Our assessment used existing facility data from a large and broad selection of health-care facilities specializing in maternity services in 10 countries in Africa and Asia to assess the availability of data for each indicator (and the variability in data availability). Our work demonstrated that, while some of the proposed indicators can already be applied, other indicators need to be refined or will need additional sources and methods of data collection.

WHO indicators for quality of maternal health care (M1–M6), are related to clinical process, and require observation or special recording, and are unlikely to be captured in full as part of a standard facility survey. Sampling of case records and registers could be used to make the indicators more appropriate for measurement of the quality of the services provided. The indicators of quality of newborn care (N1‒N5) include composite indicators (e.g. essential care at birth) which are in practice challenging to define and capture. The denominators for some of the indicators vary, and encompass mothers and babies as well as facilities, which allows for capturing a wide range of information. In practice, however, a variety of denominators may complicate any attempt to collect data in a standardized manner that allow for comparison across health-care facilities or geographical settings.^13^

Indicators that measure coverage of care and policy or guideline adherence require additional information to be useful for monitoring of quality of care. For example, whether women with pre-eclampsia and eclampsia are treated with magnesium sulfate (indicator M2) is not routinely recorded in registers, and may require analysis of case notes. Likewise, information on time oxytocin was administered (indicator M3) is not routinely recorded, and the 1-minute timeframe may not be realistic. For the indicators on essential newborn care (N2) and kangaroo care (N3), no standardized data are currently collected in registers or case notes, and so monitoring of these indicators would require new or modified data collection tools.

In terms of further specifications required, some indicators need further work to operationalize them. For example, standards are needed to clarify the meaning of terms such as operational (for indicator N3), prolonged labour (for indicator M4) and severe systemic infection (for indicator M6) and to agree clear definitions and criteria for terminology to ensure that they can be effectively utilized for comparison across countries. Other indicators require specifying so that it is clear what needs to be captured in a way that would be measurable. For example, if both early and late neonatal mortality are to be included in health facilities’ recording of the neonatal mortality rate by birth weight (indicator N4), then there is a need to collect community data or for functioning vital registration systems to be in place.^27^^,^^28^

In general, the proposed indicators also need to include a specified timeframe for evaluation, e.g. per quarter, in line with UN facility survey standards.^23^^,^^29^ This is probably particularly pertinent for measuring stock-outs of essential drugs (indicator G1), but would be helpful for standardizing data collection for other indicators. For stock-outs of drugs, it would be helpful to differentiate between time-bound and permanent lack of availability of products or services. This could mean recording whether the drug was only temporarily unavailable and defining the number of days before a temporary lack of drugs is classified as a stock-out. Additionally, the list of essential drugs needs to take into account regional or local guidelines and practices, and could include a tracer drug or drugs (at least one of which needs to be available) to allow for standard monitoring.

Better data are needed with regard to both the availability of maternal and newborn care and the equality of that care. For the proposed indicators to provide an assessment of quality, not just coverage, of care they will need to reflect all components of care provision including input, process and outcome measures.^30^ Moreover, quality of care can mean different things to the provider and the consumer of care.^31^^,^^32^ The current set of proposed indicators does include input, process and outcome measures and is therefore a useful basis for assessing care. Nevertheless, the list will need further refinement and possibly expansion to ensure that the indicators used are representative of all aspects of quality.

This study had some limitations. First, the data used in the analysis were not collected for the purpose of this project and therefore some aspects of the assessment could not be performed. Second, the findings may not be generalizable as the results may not necessarily reflect the situation nationally in the countries from which the data originated or may not be immediately applicable to other settings. On the other hand, the data covered 10 countries in Africa and Asia and the national data recording systems within these countries are uniform. We argue that facility records can be a source of robust evidence when indicators are clearly defined and specified in existing registers.

Overall, the WHO proposed global core indicators focus on important elements of quality of care around the time of birth, and of care of the small or sick newborn, and include a balance of intervention coverage, process of care and impact indicators. However, several of the proposed indicators require some revision to be applied in real-world settings for measuring care in health facilities. In addition, for the indicators that measure coverage of care or availability of services or products, there is a need to further strengthen measurement of care quality. Collecting additional information which is not captured routinely at facilities is challenging in large-scale surveys.
